# 2-[4-(Methyl­sulfon­yl)phen­yl]acetonitrile

**DOI:** 10.1107/S1600536811003837

**Published:** 2011-02-05

**Authors:** Hoong-Kun Fun, Ching Kheng Quah, V. Sumangala, D. Jagadeesh Prasad, Boja Poojary

**Affiliations:** a X-ray Crystallography Unit, School of Physics, Universiti Sains Malaysia, 11800 USM, Penang, Malaysia; b Department of Chemistry, Mangalore University, Mangalagangotri, Karnataka State 574 199, India

## Abstract

In the title compound, C_9_H_9_NO_2_S, the benzene ring and the acetonitrile group are approximately coplanar, with a C—C—C—C torsion angle of 1.1 (3)° between them. In the crystal, mol­ecules are linked *via* inter­molecular C—H⋯O hydrogen bonds into layers parallel to (001).

## Related literature

For general background to and the biological activity of COX-2 inhibitors, see: Orjales *et al.* (2008 ▶); Zarghi *et al.* (2008 ▶); Shah *et al.* (2010 ▶); Arico *et al.* (2002 ▶); Davies *et al.* (2002 ▶); Sawaoka *et al.* (1998 ▶); Liu *et al.* (2000 ▶); Pasinetti (2001 ▶); Norman *et al.* (1995 ▶). For a related structure, see: Charlier *et al.* (2004 ▶). For bond-length data, see: Allen *et al.* (1987 ▶).
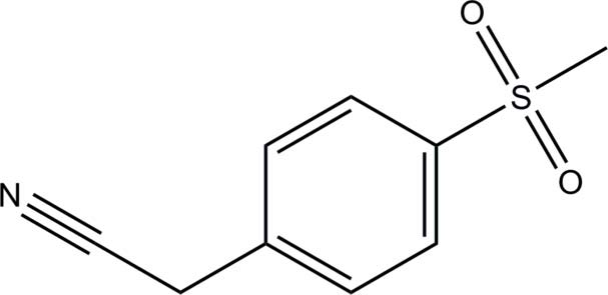


## Experimental

### 

#### Crystal data

C_9_H_9_NO_2_S
*M* *_r_* = 195.23
Triclinic, 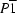

*a* = 5.5599 (2) Å
*b* = 8.0942 (3) Å
*c* = 10.9006 (4) Å
α = 81.162 (2)°
β = 85.347 (2)°
γ = 74.458 (2)°
*V* = 466.60 (3) Å^3^
*Z* = 2
Mo *K*α radiation
μ = 0.31 mm^−1^
*T* = 296 K
0.51 × 0.28 × 0.14 mm

#### Data collection

Bruker SMART APEXII CCD area-detector diffractometer
Absorption correction: multi-scan (*SADABS*; Bruker, 2009 ▶) *T*_min_ = 0.810, *T*_max_ = 0.957
5970 measured reflections
1826 independent reflections
1673 reflections with *I* > 2σ(*I*)
*R*_int_ = 0.023

#### Refinement

*R*[*F* ^2^ > 2σ(*F* ^2^)] = 0.038
*wR*(*F* ^2^) = 0.110
*S* = 1.09
1826 reflections
119 parameters
H-atom parameters constrained
Δρ_max_ = 0.29 e Å^−3^
Δρ_min_ = −0.42 e Å^−3^

### 

Data collection: *APEX2* (Bruker, 2009 ▶); cell refinement: *SAINT* (Bruker, 2009 ▶); data reduction: *SAINT*; program(s) used to solve structure: *SHELXTL* (Sheldrick, 2008 ▶); program(s) used to refine structure: *SHELXTL*; molecular graphics: *SHELXTL*; software used to prepare material for publication: *SHELXTL* and *PLATON* (Spek, 2009 ▶).

## Supplementary Material

Crystal structure: contains datablocks global, I. DOI: 10.1107/S1600536811003837/is2671sup1.cif

Structure factors: contains datablocks I. DOI: 10.1107/S1600536811003837/is2671Isup2.hkl

Additional supplementary materials:  crystallographic information; 3D view; checkCIF report

## Figures and Tables

**Table 1:** Hydrogen-bond geometry (Å, °)

*D*—H⋯*A*	*D*—H	H⋯*A*	*D*⋯*A*	*D*—H⋯*A*
C5—H5*A*⋯O1^i^	0.93	2.47	3.384 (2)	169
C9—H9*B*⋯O2^ii^	0.96	2.39	3.343 (3)	175

Symmetry codes: (i) 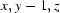; (ii) 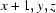.

**Fig. 1.**
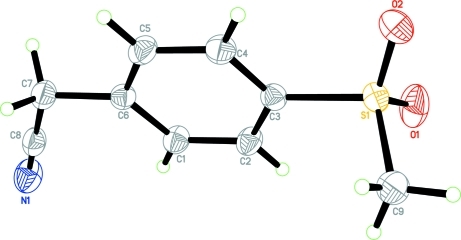
The molecular structure of the title compound showing 30% probability displacement ellipsoids for non-H atoms and the atom-numbering scheme.

**Fig. 2.**
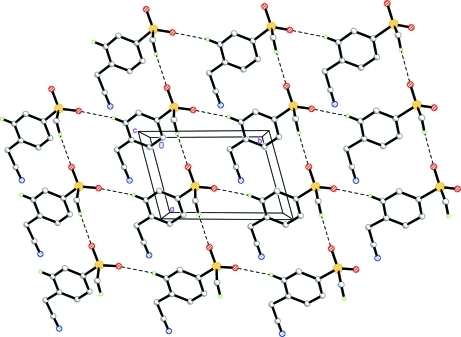
The crystal structure of the title compound, viewed along the c axis. H atoms not involved in hydrogen bonds (dashed lines) have been omitted for clarity.

## supplementary crystallographic information

### Comment

Compounds bearing the 4-methylsulfonylphenyl moiety are found to possess diverse biological properties. They are found to be highly potent and specific COX-2 inhibitors (Orjales *et al.*, 2008; Zarghi *et al.*, 2008; Shah *et al.*, 2010). Recent studies have shown that selective COX-2 inhibitors can induce apoptosis in colon, stomach, prostate, and breast cancer cell lines (Arico *et al.*, 2002; Davies *et al.*, 2002; Sawaoka *et al.*, 1998; Liu *et al.*, 2000). Selective COX-2 inhibitors offer potential treatment for the prophylactic prevention of inflammatory neurodegerative disorders such as Alzheimer's disease (Pasinetti, 2001). They are also found to be anti-inflammatory agents (Norman *et al.*, 1995). The crystal structure of a methylsulfonylphenyl derivative has been reported (Charlier *et al.*, 2004).

The molecular structure is shown in Fig. 1. Bond lengths (Allen *et al.*, 1987) and angles are within normal ranges. The benzene ring (C1–C6) and the acetonitrile group (C7/C8/N1) are approximately coplanar [torsion angles C1—C6—C7—C8 = 1.1 (3) and C5—C6—C7—C8 = -178.67 (16) °]. In the crystal packing (Fig. 2), the molecules are linked *via* intermolecular C5–H5A···O1 and C9–H9B···O2 (Table 1) hydrogen bonds into infinite two-dimensional planes parallel to (001).

### Experimental

4-Methylthiophenylacetonitrile (0.1 mol) was taken in 3 mL of acetic anhydride and cooled to 5°C. To the reaction mixture sodium tungstate (0.02 mol) was added followed by 30% hydrogen peroxide (0.2 mol) in 1.2 mL of acetic acid and water mixture (in 2:1 ratio). The temperature of the reaction mixture was slowly brought to room temperature. The completion of reaction was monitored by TLC. The solid precipitate was filtered and washed with water until the pH became neutral. The product was dried at 65 °C for 10-12 h. The product was then recrystallized in methanol (*m. p.*: 120–124 °C).

### Refinement

All H atoms were positioned geometrically and refined using a riding model with C—H = 0.93–0.97 Å and *U*_iso_(H) = 1.2 or 1.5 *U*_eq_(C). The highest residual electron density peak is located at 0.88 Å from C3 and the deepest hole is located at 0.74 Å from S1. A rotating-group model was applied for the methyl group.

### Crystal data

**Table:**

C_9_H_9_NO_2_S	*Z* = 2
*M**_r_* = 195.23	*F*(000) = 204
Triclinic, *P*1	*D*_x_ = 1.390 Mg m^−^^3^
Hall symbol: -P 1	Mo *K*α radiation, λ = 0.71073 Å
*a* = 5.5599 (2) Å	Cell parameters from 4240 reflections
*b* = 8.0942 (3) Å	θ = 2.6–33.0°
*c* = 10.9006 (4) Å	µ = 0.31 mm^−^^1^
α = 81.162 (2)°	*T* = 296 K
β = 85.347 (2)°	Block, colourless
γ = 74.458 (2)°	0.51 × 0.28 × 0.14 mm
*V* = 466.60 (3) Å^3^	

### Data collection

**Table:**

Bruker SMART APEXII CCD area-detector diffractometer	1826 independent reflections
Radiation source: fine-focus sealed tube	1673 reflections with *I* > 2σ(*I*)
graphite	*R*_int_ = 0.023
φ and ω scans	θ_max_ = 26.0°, θ_min_ = 1.9°
Absorption correction: multi-scan (*SADABS*; Bruker, 2009)	*h* = −6→6
*T*_min_ = 0.810, *T*_max_ = 0.957	*k* = −9→9
5970 measured reflections	*l* = −13→13

### Refinement

**Table:**

Refinement on *F*^2^	Primary atom site location: structure-invariant direct methods
Least-squares matrix: full	Secondary atom site location: difference Fourier map
*R*[*F*^2^ > 2σ(*F*^2^)] = 0.038	Hydrogen site location: inferred from neighbouring sites
*wR*(*F*^2^) = 0.110	H-atom parameters constrained
*S* = 1.09	*w* = 1/[σ^2^(*F*_o_^2^) + (0.054*P*)^2^ + 0.1771*P*] where *P* = (*F*_o_^2^ + 2*F*_c_^2^)/3
1826 reflections	(Δ/σ)_max_ = 0.001
119 parameters	Δρ_max_ = 0.29 e Å^−^^3^
0 restraints	Δρ_min_ = −0.42 e Å^−^^3^

### Special details

**Table:**

Geometry. All esds (except the esd in the dihedral angle between two l.s. planes) are estimated using the full covariance matrix. The cell esds are taken into account individually in the estimation of esds in distances, angles and torsion angles; correlations between esds in cell parameters are only used when they are defined by crystal symmetry. An approximate (isotropic) treatment of cell esds is used for estimating esds involving l.s. planes.
Refinement. Refinement of F^2^ against ALL reflections. The weighted R-factor wR and goodness of fit S are based on F^2^, conventional R-factors R are based on F, with F set to zero for negative F^2^. The threshold expression of F^2^ > 2sigma(F^2^) is used only for calculating R-factors(gt) etc. and is not relevant to the choice of reflections for refinement. R-factors based on F^2^ are statistically about twice as large as those based on F, and R- factors based on ALL data will be even larger.

### Fractional atomic coordinates and isotropic or equivalent isotropic displacement parameters (Å^2^)

**Table:**

	*x*	*y*	*z*	*U*_iso_*/*U*_eq_	
S1	0.64766 (8)	0.30347 (6)	0.36428 (4)	0.04502 (19)	
O1	0.6714 (4)	0.45775 (18)	0.28743 (16)	0.0728 (5)	
O2	0.4011 (3)	0.2889 (2)	0.40261 (19)	0.0773 (5)	
N1	1.5750 (4)	−0.2897 (2)	−0.02043 (19)	0.0638 (5)	
C1	1.1353 (3)	−0.0135 (2)	0.15700 (17)	0.0427 (4)	
H1A	1.2799	−0.0114	0.1081	0.051*	
C2	1.0162 (3)	0.1282 (2)	0.21644 (17)	0.0429 (4)	
H2A	1.0801	0.2246	0.2080	0.051*	
C3	0.8008 (3)	0.1237 (2)	0.28841 (15)	0.0365 (4)	
C4	0.7055 (3)	−0.0194 (2)	0.30170 (17)	0.0439 (4)	
H4A	0.5607	−0.0212	0.3505	0.053*	
C5	0.8260 (3)	−0.1598 (2)	0.24231 (18)	0.0445 (4)	
H5A	0.7620	−0.2561	0.2512	0.053*	
C6	1.0422 (3)	−0.1580 (2)	0.16938 (15)	0.0370 (4)	
C7	1.1679 (4)	−0.3160 (2)	0.10645 (18)	0.0464 (4)	
H7A	1.2073	−0.4167	0.1694	0.056*	
H7B	1.0511	−0.3343	0.0517	0.056*	
C8	1.3966 (4)	−0.3021 (2)	0.03441 (18)	0.0468 (4)	
C9	0.8180 (4)	0.2777 (3)	0.4977 (2)	0.0586 (5)	
H9A	0.7482	0.3732	0.5433	0.088*	
H9B	0.9891	0.2744	0.4738	0.088*	
H9C	0.8100	0.1716	0.5490	0.088*	

### Atomic displacement parameters (Å^2^)

**Table:**

	*U*^11^	*U*^22^	*U*^33^	*U*^12^	*U*^13^	*U*^23^
S1	0.0405 (3)	0.0414 (3)	0.0506 (3)	−0.00199 (19)	−0.00442 (19)	−0.0129 (2)
O1	0.1054 (14)	0.0367 (8)	0.0691 (10)	−0.0059 (8)	−0.0101 (9)	−0.0042 (7)
O2	0.0381 (8)	0.0876 (12)	0.1116 (14)	−0.0088 (7)	0.0091 (8)	−0.0496 (11)
N1	0.0653 (12)	0.0591 (11)	0.0703 (12)	−0.0208 (9)	0.0182 (10)	−0.0217 (9)
C1	0.0428 (9)	0.0427 (10)	0.0458 (10)	−0.0169 (7)	0.0069 (7)	−0.0099 (7)
C2	0.0466 (10)	0.0382 (9)	0.0479 (10)	−0.0185 (7)	0.0028 (8)	−0.0079 (7)
C3	0.0367 (8)	0.0361 (8)	0.0354 (8)	−0.0072 (7)	−0.0024 (6)	−0.0042 (6)
C4	0.0390 (9)	0.0468 (10)	0.0474 (10)	−0.0153 (8)	0.0042 (7)	−0.0070 (8)
C5	0.0471 (10)	0.0393 (9)	0.0519 (10)	−0.0200 (8)	−0.0002 (8)	−0.0062 (8)
C6	0.0395 (9)	0.0366 (8)	0.0351 (8)	−0.0094 (7)	−0.0051 (7)	−0.0050 (7)
C7	0.0500 (10)	0.0394 (9)	0.0516 (11)	−0.0119 (8)	−0.0004 (8)	−0.0123 (8)
C8	0.0567 (12)	0.0381 (9)	0.0463 (10)	−0.0094 (8)	−0.0016 (9)	−0.0138 (8)
C9	0.0550 (12)	0.0689 (14)	0.0505 (11)	−0.0037 (10)	−0.0073 (9)	−0.0231 (10)

### Geometric parameters (Å, °)

**Table:**

S1—O1	1.4239 (16)	C4—C5	1.381 (3)
S1—O2	1.4306 (16)	C4—H4A	0.9300
S1—C9	1.755 (2)	C5—C6	1.388 (2)
S1—C3	1.7657 (17)	C5—H5A	0.9300
N1—C8	1.136 (3)	C6—C7	1.519 (2)
C1—C6	1.385 (2)	C7—C8	1.461 (3)
C1—C2	1.387 (2)	C7—H7A	0.9700
C1—H1A	0.9300	C7—H7B	0.9700
C2—C3	1.383 (2)	C9—H9A	0.9600
C2—H2A	0.9300	C9—H9B	0.9600
C3—C4	1.382 (3)	C9—H9C	0.9600
			
O1—S1—O2	117.73 (12)	C4—C5—H5A	119.8
O1—S1—C9	108.35 (11)	C6—C5—H5A	119.8
O2—S1—C9	108.32 (12)	C1—C6—C5	119.12 (16)
O1—S1—C3	108.95 (9)	C1—C6—C7	122.56 (16)
O2—S1—C3	108.26 (9)	C5—C6—C7	118.32 (15)
C9—S1—C3	104.42 (9)	C8—C7—C6	113.81 (15)
C6—C1—C2	120.95 (16)	C8—C7—H7A	108.8
C6—C1—H1A	119.5	C6—C7—H7A	108.8
C2—C1—H1A	119.5	C8—C7—H7B	108.8
C3—C2—C1	118.95 (16)	C6—C7—H7B	108.8
C3—C2—H2A	120.5	H7A—C7—H7B	107.7
C1—C2—H2A	120.5	N1—C8—C7	179.0 (2)
C4—C3—C2	120.80 (16)	S1—C9—H9A	109.5
C4—C3—S1	119.91 (13)	S1—C9—H9B	109.5
C2—C3—S1	119.28 (13)	H9A—C9—H9B	109.5
C5—C4—C3	119.72 (16)	S1—C9—H9C	109.5
C5—C4—H4A	120.1	H9A—C9—H9C	109.5
C3—C4—H4A	120.1	H9B—C9—H9C	109.5
C4—C5—C6	120.45 (16)		
			
C6—C1—C2—C3	−0.3 (3)	C2—C3—C4—C5	−0.1 (3)
C1—C2—C3—C4	0.3 (3)	S1—C3—C4—C5	−179.93 (13)
C1—C2—C3—S1	−179.94 (13)	C3—C4—C5—C6	0.0 (3)
O1—S1—C3—C4	−145.12 (16)	C2—C1—C6—C5	0.1 (3)
O2—S1—C3—C4	−15.97 (18)	C2—C1—C6—C7	−179.65 (16)
C9—S1—C3—C4	99.29 (17)	C4—C5—C6—C1	0.0 (3)
O1—S1—C3—C2	35.06 (17)	C4—C5—C6—C7	179.80 (16)
O2—S1—C3—C2	164.22 (15)	C1—C6—C7—C8	1.1 (3)
C9—S1—C3—C2	−80.53 (17)	C5—C6—C7—C8	−178.67 (16)

### Hydrogen-bond geometry (Å, °)

**Table:**

*D*—H···*A*	*D*—H	H···*A*	*D*···*A*	*D*—H···*A*
C5—H5A···O1^i^	0.93	2.47	3.384 (2)	169
C9—H9B···O2^ii^	0.96	2.39	3.343 (3)	175

Symmetry codes: (i) *x*, *y*−1, *z*; (ii) *x*+1, *y*, *z*.
